# Older Adults’ Experiences of Institutional Eating and Dining: A Qualitative Study on Mealtimes in Adult Day Centers

**DOI:** 10.3390/nu17030420

**Published:** 2025-01-23

**Authors:** Rinat Avraham, Natan Lev, Jonathan M. Deutsch, Nadav Davidovitch, Stav Shapira

**Affiliations:** 1 Recanati School for Community Health Professions, Department of Nursing, Faculty of Health Sciences, Ben-Gurion University of the Negev, Beer Sheva 84101, Israel; 2 School of Public Health, Faculty of Health Sciences, Ben-Gurion University of the Negev, Beer Sheva 84105, Israel; levnat@post.bgu.ac.il (N.L.); nadavd@bgu.ac.il (N.D.); stavshap@bgu.ac.il (S.S.); 3 Department of Health Sciences, College of Nursing and Health Professions, Drexel University, Philadelphia, PA 19104, USA; st96d633@drexel.edu

**Keywords:** adult day centers, healthy aging, healthy placemaking, mealtime experience, older adults, qualitative research

## Abstract

**Background/Objectives:** As the global population ages, it is becoming increasingly important to create sustainable, health-promoting environments that support healthy aging. This study explores seniors’ mealtime experiences in adult day centers (ADCs) in southern Israel, focusing on identifying health and well-being needs related to eating and dining behaviors through the lens of the healthy placemaking approach. **Methods:** Thematic analysis was used to analyze data from focus groups and interviews with ADC attendees and leaders across a multicultural sample of ADCs in southern Israel between April and November 2022. **Results:** Three main themes emerged from the study: (1) individual-level needs, which are met through meals or during mealtimes and include positive experiences, a sense of empowerment, and the cultivation of warmth and domesticity; (2) social needs, which are addressed through interactions during mealtimes and food-related behaviors, including building social connections, fostering community, and encouraging social engagement; and (3) sustainability, health, and environmental aspects, including promoting a healthy and disease-appropriate diet, alongside addressing ecological and food security concerns. **Conclusions:** We demonstrate the pivotal role of ADC meals in facilitating social engagement and fostering a sense of community among attendees. Additionally, we highlight the importance of centering attendees’ concerns and needs in the dining experience and promoting their active participation in decision-making processes. Transforming ADC meals through the healthy placemaking approach can promote healthy eating, enhance social interactions, and support sustainable environments.

## 1. Introduction

The significant global increase in life expectancy is a remarkable achievement of modern civilization. This upward trend in longevity has led to a greater focus on senior citizens’ quality of life, along with increasing awareness that social and healthcare systems need to expand their services to meet the needs of a growing older adult population, promote their social engagement, and develop new models of care delivery [1]. Healthy older adults are valuable to their families, communities, and society as a whole [2]. However, this demographic shift of rising numbers of older adults also presents various challenges, such as the increased prevalence of chronic, age-related diseases [3] and a deterioration in independence and social participation [4]. Many of these challenges can be prevented or alleviated through lifestyle modifications, including dietary changes and physical activity [5], as well as interventions addressing loneliness and social isolation [6,7].

Community-based care facilities for older adults, such as adult day centers (ADC), are essential in providing the care and support necessary for this age cohort, as these facilities help to prevent illness and promote healthy and successful aging [8]. ADCs typically utilize community settings and are designed to cater to independent or semi-independent senior citizens who do not require intensive supervision or assistance. ADCs aim to prevent isolation and slow cognitive and physical decline among older adults living in the local community by providing a supportive, professionally staffed environment. Additionally, ADCs serve as a convenient platform for delivering various programs that aim to address the nutritional, daily living, and social needs of older adults [9].

ADCs in Israel, under the supervision of the Ministry of Welfare and Social Affairs, are intended for senior citizens residing in the community who do not require constant supervision or assistance but may experience feelings of loneliness or a lack of family support. Alongside addressing this loneliness, ADCs assist in key areas, such as ensuring proper nutrition and enriching leisure time with social, cultural, and other engaging activities [10]. In this regard, mealtimes—which are a central part of the daily routine in ADCs—provide ample opportunities to address all of these areas.

Beyond the obvious goal of facilitating the consumption of food and drink, mealtimes offer opportunities for conversation and companionship [11]. Indeed, mealtimes in ADCs and other long-term care settings have been investigated in relation to nutritional parameters and have been found to positively impact attendees’ energy, protein, and fluid intake [12,13]. Concurrently, other studies have investigated how mealtime experiences, communal dining, and dining room enhancements affect social health and well-being. For example, Bjørner et al. examined contextual factors that can positively affect mealtimes among Danish older adults living at home. Using food diaries, documentary photos, and in-depth interviews, the authors demonstrated the major role that social factors, such as eating with family and friends, play in positive mealtime experiences [14].

Similarly, Morrison-Koechl et al. identified the positive impact of social engagement on the food energy consumption of residents in long-term care homes for older adults. Using observations, structured interviews, and nutritional measures, Morrison-Koechl et al. found an indirect association between low social engagement and lower food energy intake. They, therefore, surmised that providing more positive social interactions during meals may improve the quality of life and slow the decline of residents [15]. These studies highlight the importance of socializing during mealtimes and its contribution to improved quality of life and appetite.

Likewise, a systematic review conducted by Watkins et al. explored attitudes, perceptions, and mealtime experiences among staff and residents in adult care homes. By synthesizing qualitative data from interview studies, they identified four main themes that affect the nutritional status, health, and well-being of residents: support offered during the meal by staff, individual autonomy of residents during the meal, social and cultural aspects of the meal, and physical elements of the meal (food quality and variety, dining room environment, and so on) [11]. These diverse themes underscore that, in order to improve residents’ mealtime experiences and overall health, multifaceted interventions that address meal quality, mealtime culture, and resident autonomy are needed, rather than single-component solutions, such as enhancing meal quality or dining décor alone. A similar multifaceted approach is the focus of the present study, which aims to highlight the practical aspects of redesigning and improving mealtime experiences in ADCs in a way that promotes autonomy, health, and well-being while also emphasizing the unique perspectives of attendees.

We draw upon the theoretical approach of healthy placemaking, which emphasizes the creation of environments that foster well-being, safety, and sustainability [16]. These environments often feature physical elements such as access to green spaces and improved walkability, but they also encompass broader aspects, such as healthy eating [17]. Healthy placemaking focuses on changing the way we live and the places we live in by creating healthier, happier, safer, and more sustainable environments [18,19,20]. It encourages creative patterns of health promotion activities while integrating not only the urban and physical design but also cultural and social aspects that shape a place and support its ongoing development in ways that promote health and happiness. In the context of older people, healthy placemaking emphasizes the importance of creating meaningful environments that foster a sense of belonging, autonomy, independence, safety, and security, which are critical to well-being [21,22].

Recent evidence draws attention to the impact of public and communal places, such as ADCs, on successful aging. It highlights their role in supporting the “aging in place” paradigm, with studies showing that such spaces are crucial for enabling social participation and maintaining physical health [23,24] while also facilitating health equity [25]. When applied to the context of mealtimes in ADCs, healthy placemaking involves designing dining experiences that not only prioritize physical accessibility (i.e., adapting environments based on progressive disability) and nutritious food options but also enhance social engagement, personal autonomy, and sustainability. One key aspect of healthy placemaking is the creation of codesigned environments that involve participants as active contributors. This participatory approach enables individuals to shape their surroundings based on their unique needs, habits, and preferences, which, in turn, enhances both their physical and mental health, as well as their overall well-being. Engaging older adults in the design process is particularly important, as such involvement fosters a sense of ownership and belonging, leading to environments that better support their autonomy and social integration [22,26].

The present study explores the perceptions of ADC attendees in the Negev region of Israel regarding the institutional catering provided at these centers. Grounded in the principles of the healthy placemaking approach, it delves into the perspectives of older adults in order to assess their satisfaction and explore the significance of food and dining experiences in daycare settings. Specifically, this study identifies food- and dining-related behaviors and attitudes of ADC attendees, along with their specific health and well-being needs associated with eating and dining behaviors in these centers.

## 2. Materials and Methods

### 2.1. Design and Setting

This study was planned and carried out in ADCs managed by local authorities in the southern district of Israel. This area, which covers approximately 60% of the country’s land surface, features a wide range of settlement types, from rural to urban, and is home to a culturally diverse population that includes Bedouins, Jews, and immigrants. We employed a qualitative approach using the thematic analysis methodology [27]. This methodology is flexible and appropriate when the aim is to understand participants’ meaning in relation to real life through the investigation of lived experience—a first-hand perspective that requires deep reflection to understand and formulate. Focus groups and interviews are appropriate qualitative methods for this investigation type, as they enable people to describe experiences stemming from their participation in real-life experiences [28]. Thus, our study comprised both focus groups with ADC attendees and semi-structured interviews with ADC staff.

### 2.2. Participants and Recruitment

We conducted seven focus group interviews (*n* = 28) in the ADCs, with 2–5 ADC participants in each group. We also conducted semi-structured interviews with ADC staff (*n* = 7). All focus group participants were ADC attendees who were both physically and mentally self-sufficient. As ADCs tend to cater to ethnic populations and may be exclusively for women or men, we used a purposive sampling method, recruiting a diverse sample based on ADC attendees’ characteristics, including gender, religion, culture, and housing situation [29]. Participation in the study was voluntary, with interviews being open to any interested individuals. In addition, the ADCs’ staff members helped us to identify and approach potential participants who they anticipated would feel comfortable talking freely and who showed interest in the study topics (i.e., rich informants). Interviews with ADC leadership were conducted before or after the focus groups in the same centers from which attendees were recruited. Both the focus groups and the interviews took place on the days when the centers were operational—specifically, during the intervals between meals. Table 1 presents the characteristics of attendees in each focus group.

### 2.3. Procedure

We developed an interview guide for the focus groups with ADC attendees and for personal interviews with on-the-ground leadership. The creation of this guide was informed by a comprehensive consultation process involving experts from multiple disciplines. We sought feedback from specialists in the Department of Senior Citizens at the Israeli Ministry of Welfare and Social Affairs, who provided insights into policy relevance (i.e., what is the ministry policies and regulations regarding meals and food serving in the ADC) and the specific needs of older adults. Environmental psychologists contributed by ensuring that the guide adequately addressed the psychosocial and environmental factors influencing behavior in communal settings. Additionally, experts in qualitative research methods, particularly those with expertise in food and hospitality both in Israel and abroad, were consulted to ensure the guide’s rigor and relevance to the study’s context. These experts were asked to review the content, structure, and wording of the guide to verify that it would effectively capture the nuanced experiences of participants. Their detailed feedback led to refinements in the questions, phrasing, and overall flow of the guide; this helped to ensure clarity, cultural appropriateness, and the potential for generating meaningful data. The research team finalized the interview guide based on their collective input.

Prior to commencing the study, the research team visited the participating ADCs to become acquainted with the leaders and attendees and present the study’s aims and procedures to them. Following each visit, a date was coordinated with the staff to conduct both the focus groups and the semi-structured interviews in person. If staff members were not available during the center’s activity time, interviews were scheduled at a time convenient for both parties, sometimes taking place via telephone.

Focus groups were structured as follows: They began with an introductory portion in which attendees were welcomed and received instructions pertaining to the focus group and an estimated timeframe for its completion. An opening question was then employed to break the ice and help participants feel at ease. The main part of the focus group was divided into three topics of discussion: (1) participants’ eating and dining goals and habits; (2) the experiences and meanings of meals in the ADC; and (3) participants’ views of potential directions for action to help improve mealtimes in the ADC. After concluding the main part, participants were given the option to freely raise any additional issues they wanted to talk about. The focus groups were concluded with an expression of thanks and gratitude from the researchers for the time and effort invested by the participants. Each focus group lasted between 90 and 120 min.

Interviews with leaders were conducted based on five leading questions: (1) “Tell me about the ADC you’re in charge of. Who are the attendees and what characterizes them?”; (2) “Tell me about the meals at the center. How are they conducted?”; (3) “Tell me about the food served at the center. Who prepares it, where does it come from, and what do the attendees think of it?”; (4) “Do the centers’ attendees participate in meal preparation? If so, how?”; and (5) “If you could, what would you change about the meals in your ADC?” Each personal interview with the ADC leaders lasted between 45 and 60 min.

Interviews and focus groups were conducted in pairs, either by two researchers from the study group (A.R. and S.S.) or by two students who received proper instruction and training to ensure consistency in the interviewing style [30]. Before the interviews began, participants received a detailed explanation of the interview structure and objectives and then signed an informed consent form. During the interviews, one interviewer led the discussion while the other took notes and kept track of the timeframe. Interviews were audio-recorded with express permission from the interviewees.

### 2.4. Data Analysis

Data from the audiotaped interviews were transcribed verbatim using Express Scribe Transcription Software (Version 5.84, NCH Software, Greenwood Village, CO, USA). Analysis using MAXQDA software (version 24, Cleverbridge GmbH, Cologne, Germany) was conducted by two independent researchers based on the tradition of thematic analysis. The two researchers read through the data from the interviews several times to ensure that they understood the meaning of each response. Then, they performed a thematic analysis to validate their interpretation of the first step. This included structuring the text into meaning units, then categorizing groups of meaning units under subcategories, and finally organizing them under the main themes. They reached a comprehensive understanding of the meaning by reflecting the results onto the relevant literature. The researchers discussed the results after each step to resolve disagreements and to ensure the reliability of the interpretation throughout the entire process.

## 3. Results

The content analysis incorporated interview information obtained from the focus group participants and ADC leaders.

**Figure 1:**
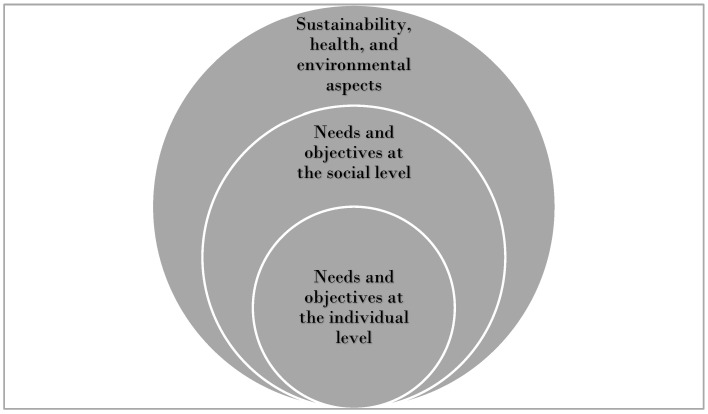
Relationships among emerging themes.

In line with the socio-ecological model [31], we suggest that the meal and food experiences at ADCs have three levels of impact: (1) individual; (2) social; and (3) sustainability, health, and environmental. For each level, we identified specific needs and objectives. While three distinct themes were identified, there are also overlaps between them that indicate shared objectives between levels, as reflected in Figure 1.

Three main themes emerged from the collected data, reflecting the goals and needs perceived by attendees and leaders, as well as their beliefs concerning the meaning of eating and dining at the center. The codes, categories, and themes identified are summarized in Table 2 and described below, together with exemplifying quotes.

### 3.1. Theme 1: Needs and Objectives at the Individual Level

The first theme revolves around fulfilling individual needs and objectives through the ADC’s meals. These needs relate to the dining experience, the taste and enjoyment of the food, and feelings of competency and independence experienced by attendees.

A prominent category derived from individual needs is the expectation that food served at the ADC will provide positive experiences. Attendees anticipate mealtimes as a pleasurable experience, appreciating the food not only for its nutritional value but also for the gustatory pleasure it provides. This underscores the importance of the food’s freshness and taste in enhancing the mealtime experience. The positive experiences of older adults from typical daily activities, such as eating, can reflect meaningful factors related to their overall well-being—for example, sensory enjoyment derived from the taste, smell, and texture that stimulates pleasure and satisfaction. This sensory stimulation may become especially significant as other sources of joy decrease with age. This is reflected in the following quote (respondent codes are derived from the respondent’s first initial and the ADC number):

Food is fun. It’s important to me that it will be tasty. When it doesn’t taste good, I don’t enjoy it. I eat for the pleasure of it.

(E4)

Participants further emphasized the desire to eat freshly made, non-processed foods, as these are associated with health and quality and may affect both physical and emotional well-being. The smell of freshly cooked food, the sight of meals being prepared, and the presentation of dishes can stimulate appetite, which can be particularly important for older adults who may experience reduced appetite due to aging, medications, or health conditions. A5 noted a distinct preference for meals prepared on-site:

Lunch is prepared by a catering company, [but] breakfast [is made] by the workers at the ADC. The breakfast is varied and fresh. But at noon… for example, the salad, […] I don’t know when they cut it, [but] it’s not tasty! I can’t eat the salad. It’s always mushy. I love fresh salad that [has been] made *now*, and I [can] eat *now*!

(A5)

This sentiment was echoed by an instructor from another center while detailing her approach to meal preparation. She emphasized the need to pay attention to attendees’ preferences, the importance of the freshness of ingredients, and the need to make dishes familiar to attendees:

I make sure to prepare very elaborate meals for them…the women in the center also help in the preparation. I also prepare special porridges for them (today, we prepared buckwheat porridge). Everything [is] according to their taste, [and we make] foods [that are] familiar to them from Russia.

(S1)

Food often carries cultural and personal significance. Enjoying familiar flavors can evoke memories, comfort, and a connection to past experiences, reinforcing identity and continuity across the lifespan. Instructors highlighted the importance of mealtime enjoyment among attendees, emphasizing their dedication to ensuring that catering meets their preferences. They also indicated the need to respond to attendees’ feedback and to ensure that they prepare meals that are both enjoyable and to the liking of those they serve.

We get lunch from the day center. There is a cook there, and the food is usually delicious. If there are any comments, the cooks accept them with love.

(M2)

One concern frequently mentioned in the various ADCs was a lack of variety in the food offered. In some places, participants noted that the menus remained largely unchanged, with only occasional or quarterly updates, and that the same dishes were served every week in a fixed order.

I don’t have a problem with any food; I have one problem—that the same food repeats itself. So, it’s tolerable and even tasty, but it’s a bit boring. I need some diversity. I have no complaints; we have many diseases, many tastes, and you can’t make everyone satisfied. But, are we just going to be bored from now on? From the beginning of the contract, the catering company has provided only three dishes, which are arranged by day. Every day, there is a fixed dish.

(S5)

Eating a variety of foods provides a sense of novelty and excitement and makes meals more enjoyable, leading to greater emotional satisfaction and a more positive outlook on daily life. In one center, we found that attendees placed special value on the variety of foods they receive and that this variety enabled everyone to have their preferred food and enjoy the meal.

I want to point out one thing that does not exist in any [other] center: We [have] about 18 to 19 people here, [and] there are three types of food. One person does not like schnitzel, so they bring him two pieces of meat. Where else is there such a thing? So, I think the approach here is very, very personal.

(N6)

Another important finding pertains to attendees’ desire to feel cared for. While many are still able to cook for themselves, they often lack the motivation to do so when this is just for their own sake. In addition, they expressed that meals that are prepared and served by others fulfill a deep desire to feel nurtured and cared for. Some are experiencing challenges such as physical decline and loss of independence, while others face emotional struggles. The cooked meals provide them with the attention and care they may no longer feel capable of giving themselves. S5 noted the following:

[The food] doesn’t feel healthier but […] here they simply invest more in terms of variety, at home we are lazier… when I come to the ADC, I don’t worry about the cooking, I don’t get angry. It’s a kind of treat… we are spoiled.

Being served meals may communicate that others are looking out for them, reinforcing the idea that attendees are valued even if they are no longer able or motivated to cook for themselves. This is clearly reflected in a quote from A6, who noted that knowing the cook personally increases attendees’ sense of care and attention and is greatly appreciated:

Since the cooks are so connected to us, they consider each [attendee individually]. This is something that cannot be done in a catering service. There really is a lot of attention paid to everyone, including their preferences and health issues.

(A6)

Not all feedback was positive, however, as some attendees indicated that the meals lacked personal attention:

The vegetables are mainly potatoes. You feel as if they are doing you a favor by giving you food; it’s not pleasant. Recently, the soup has changed—there is carrot and zucchini, but the chicken is poor, overcooked.

(S1)

Feeling overlooked or experiencing a sense of neglect may result in a sense that one is unimportant or undervalued. Thus, negative experiences from the food served reduce attendees’ satisfaction, which in turn decreases their sense of well-being.

Another category at the individual level involves empowering experiences associated with meals at the ADC. Mealtime experiences play a crucial role in fostering a sense of value and belonging among participants. Many expressed that these occasions make them feel important and connected to others. For some, the mealtime structure provides an opportunity to fulfill their need for recognition and inclusion. One poignant example comes from an attendee who shared her feelings of exclusion due to not having a designated seat at the table, unlike other diners at the center.

I don’t have a permanent place at the table, and it really bothers me. I don’t always come because my husband is [recovering from] a stroke; [as a consequence] I don’t have a permanent place. All of the women here sit in their own place.

(Z2)

The attendees’ responses also reveal that the impact of meals at the ADC extends beyond just the food served—the meals can also meet essential individual needs when given sufficient attention. The example from Z2 underscores how not having a place at the table can amplify feelings of exclusion, insecurity, lack of belonging, or a broader sense of disconnection—all of which ADCs aim to address.

Most attendees’ involvement during mealtimes consists of serving and cleaning, with little participation in cooking or selecting the menu. While this involvement helps them feel independent and useful and gives them a sense of fulfilling their responsibilities, it also allows them to show their appreciation for the service provided by assisting with the cleaning. However, many would prefer to be more engaged in other aspects of mealtimes, possibly even in preparing the food:

We help here just like we clean and organize at home, you can’t go and leave everything behind you, doing so would be shameful, not nice. [But] no one approaches the kitchen. We are not permitted entrance! What they serve, we eat.

(S7)

For some seniors, being restricted from entering the kitchen in the ADC may evoke feelings of disability, uselessness, and being burdensome—akin to being treated like small children. This can diminish their sense of self-worth and autonomy. However, while attendees expressed interest in having greater involvement in meal preparation and more influence on decisions that affect them, they noted that this may be infeasible due to practical concerns or feelings that the staff would not be motivated to help them do so. Such exclusion may further diminish their autonomy and reinforce a sense of dependency. For example, in response to being asked whether they would like to participate in choosing the menu, S1 stated,

We would like to [participate in choosing the menu], but that would be a problem because [it is the managers of the ADC] who decide.

The third category at the individual level emphasizes the attendees’ expectations that their eating environments promote warmth and domesticity. Attendees ideally want the dining environment and food provided at the ADCs to closely resemble those at home, as this fosters feelings of care, love, and familiarity. D1 noted the following:

[The leader] prepares very elaborate meals for us. A salad rich in herbs and seasoning is prepared every day… She also prepares special food, everything according to our taste, foods we are familiar with from our country of origin.

Familiar food provides a cultural connection, evokes memories from childhood, and offers a sense of comfort and belonging. It allows seniors to reconnect with their past, reinforcing their identity and personal history. At one center, a leader detailed the strong relationships that had formed among attendees, noting the care they showed for one another around mealtimes, as if they were family: “The women in the ADC take great care of the men, especially in matters of food” (S1).

When the leader is part of the attendees’ community, there is a noticeable culture-based personal connection and deep familiarity, resulting in superior individualized care that is tailored to each person’s needs and preferences:

I prepare [food] for each [attendee] according to what they are used to and can eat. Finely chopped salad, homemade tahini, white cheese, olives, a slice of bread, or a cracker for those who don’t eat bread—I know exactly what everyone likes.

(S6)

In most centers, meals are typically eaten in the same area where other activities take place. Breakfast is served during brief, relatively calm intervals between or after activities, while lunch is often held near the end of the day, causing some attendees to hurry through their meal to catch their shuttle home. The overall atmosphere during mealtime is generally pleasant; this provides opportunities for casual conversations in some settings.

In general, there is a good atmosphere in the center. Four people sit around each table, talking a little about the food. Then we talk about the arrangements for the end of the day… I go straight home when I finish eating; there are some who stay a little after the meal, but not much because there is a shuttle that leaves here … at 12.

(E4)

However, in other ADCs, media tends to dominate attendees’ attention:

[There are] no conversations during the meal. The TV works in the background.

(A6)

It appears that attendees appreciate longer and more meaningful mealtime experiences, where they can engage in conversation and connect with one another—as often happened in their homes—to enhance the overall enjoyment of the meal.

### 3.2. Theme 2: Needs and Objectives at the Social Level

The second theme emphasizes elements related to social needs and objectives that are addressed by the meals served at ADCs. Meeting these needs involves promoting a personal sense of belonging, alleviating feelings of loneliness among attendees, and fostering a sense of community and social connection with the surrounding environment.

The first category in the social needs theme highlights food as a bonding factor that promotes relationships with others. In this regard, attendees see mealtimes at the ADCs as an opportunity to nurture existing friendships and forge new ones, particularly as they are in a stage of life in which social circles often diminish. They emphasized that social interactions are a primary reason for attending the ADC and that they value mealtimes as key occasions to facilitate these connections.

It had been a long time since I came, and they asked me to come. I have been a widow for 11 years… I said for what? I go to the gym, go swimming, and finish at 10 a.m., in no rush to go anywhere. But in the end, I was convinced and I’m happy! I meet people I don’t usually see because our occupations are different. Suddenly, I met this and that [person]… Where would I meet them if not at the center?

(A5)

Their words reflect their need to form connections and to be part of a community where people genuinely care about someone who is not there. This fosters a heightened sense of belonging, making them feel part of a cohesive group. Some ADCs also celebrate personal events; this may further reinforce attendees’ sense of belonging, making them think that they are an important part of the social fabric of the center. Traditional foods eaten during celebrations help foster a sense of community and inclusiveness, as it allows attendees to bond over shared experiences and traditions, enhancing social interactions and strengthening connections with fellow attendees.

You can bring spices from home… we bring cakes and pastries for the morning with a cup of tea. Anyone who has an event, birthday, or something… Let’s say I was happy because of the election results—I made a *Mofletta* [a traditional Maghrebi Jewish pancake]!

(S7)

One leader shared a similar perspective, relating that mealtimes are used to foster social bonds and build a sense of community by encouraging attendees to share their personal celebrations with fellow ADC members. By allowing them to bring home-cooked food to the center, the leaders not only enhance attendees’ sense of enjoyment and accomplishment but also create opportunities for shared experiences and lasting memories:

Many times the breakfast buffet includes foods that attendees prepare and bring themselves to honor the members, such as jams, cakes, cookies, and more. Bringing food to the center is also used to mark personal joys as a birth of a grandchild, or a birthday) or collective holidays joys. Attendees love to celebrate and make each other happy in this way.

(S7)

Attendees are also driven by the need to alleviate their feelings of loneliness and boredom. Most are at a stage in life where they still live independently but do not have a permanent partner to share their time and meals with and therefore often eat their meals alone. As a result, they place significant value on seemingly simple opportunities for social interaction, such as sitting around the table to chat and snack together. G2 told us the following:

I usually eat alone, and here I have the opportunity to eat with friends…Look, people are sitting…chatting and snacking.

Loneliness in older age can be difficult to acknowledge; however, it is hinted at in comments like this above. Many older adults seek out experiences that differ from their routines at home, where they spend much of their time alone.

The second category in the social needs theme refers to attendees’ desire for community and social engagement. In a few cases, attendees referred to the fact that the meals at the center made them feel cherished and cared for by their community. These feelings cultivate a sense of connection to the community and to the people in their living places. They appreciate the fact that young community members value more senior ones:

Our food is subsidized. This means that our community participates 50% and the municipal authority 50%. There is no such thing anywhere else!

(N6)

These feelings of being cared for are especially significant for individuals who have spent their entire lives in the community and have actively contributed to it. They now feel that the community is “repaying” them for their contributions over the years.

I have been involved with this center for many years, not just as an elder but also when I worked for the community… I remember, and [ADC attendee] can testify since he is among the older adults, there were all kinds of methods for meal services in the center. People didn’t like the food and the repetition of the menu day after day… Then, the community developed tourism, including culinary tourism. There was a group of women who cooked, and we approached the community management and asked these women if they could take the initiative [to cook for the people in the ADC].

(A6)

In other cases, attendees told us about the significance of making food in the center for others in the community. This serves as an opportunity for them to engage in meaningful activity during their spare time while positioning themselves on the giving, rather than the receiving, side—unlike most other times in their lives. One of the leaders said the following words:

Sometimes, we prepare certain foods here for events such as parties or holidays. For example, we prepared *Sfinge* [traditional Moroccan donuts] for an event in honor of the center’s leaders. We prepared two kilos, the women here—they prepared [them], they were so proud of themselves! You won’t believe how good it makes them feel. They like to talk about it with everyone.

(M2)

When asked whether they would like to actively contribute to and engage with their communities, one participant shared her experience of volunteering in a nursing home with older adults who require extensive assistance:

I have been volunteering at the elderly home for 17 years. Once a week, I prepare food for them. For tomorrow, I have already prepared everything for them to make a Mofletta, and they are very happy that I am coming, along with another friend. To make them happy, we sing and dance with them.

(S7)

Helping others and knowing that it brings them happiness signifies that these older individuals still possess the power and ability to assist can foster joy and reduce feelings of loneliness and depression.

### 3.3. Theme 3: Sustainability, Health, and Environmental Aspects

The third theme pertains to sustainability, health, and the environmental needs and objectives of attendees in relation to the food at the ADCs. The theme encompasses goals such as maintaining a healthy and personally tailored diet, as well as achieving food security and environmental sustainability.

The first category within this theme refers to attendees’ expectations that they will be able to eat a diet that caters to their medical conditions and dietary restrictions. From the attendees’ perspective, as their daily nutrition largely depends on the meals provided at the ADC, and the food should be customized to meet these specific needs. They are aware that the food is supervised by a dietician from the Ministry of Health, and this is important to them. Since it is sometimes difficult for them to adhere to nutritional recommendations at home, they want to have the opportunity to enjoy a nutritious and balanced meal at the center.

I eat food without fat and sugar, I eat three meals a day, and I don’t eat after 5 p.m. The main meal is at noon. I don’t eat sweets and fat, but I do eat all types of meat… The meals are varied, and everyone finds what they like. It is important to me to always have bread… the food [in the ADC] feels healthy to me; if the food wasn’t tasty and healthy, I wouldn’t come to the center.

(I3)

In a sense, knowing that the food served at the center is nutritious and healthy reflects the care and consideration given both to the meals and to each individual’s specific health needs. Although attendees acknowledged the difficulties inherent in accommodating the diverse dietary needs of a population with multiple chronic conditions, they want the ADCs to serve healthy food. This is especially important as the attendees visit the centers regularly, and the impact of consuming unhealthy food over time could be harmful.

I understand that it’s limited because it’s a public meal… and you can’t make food for diabetes and food for blood pressure, and food for something else.

(S5)

Good and healthy food should be given. I would like to have no processed food, simply to put the chicken in the oven with potatoes and sweet potatoes.

(G1)

The catering company has another problem. It uses canned food and powdered food instead of fresh vegetables.

(S5)

They mentioned that, at times, they choose unhealthy food simply to curb their hunger, as there are limited nutritious options available. The following quote highlights the extent to which their nutrition relies on the meals provided at the center and the critical role that these meals play in shaping attendees’ overall health and well-being:

You get a fresh salad in a tiny bowl. I like the junk food even though it’s not healthy for me, but there is nothing else to eat… I only eat cookies when I’m here because it’s available; I don’t have it at home.

(M1)

In some instances, attendees take an active role in influencing the center’s services by stating their opinions or even choosing to replace unhealthy dishes with healthier options, again highlighting the importance they place on maintaining a healthy diet:

We decided to take out biscuits and all that stuff. Before, we would get the fruit together with lunch, but from the point of view of the dietitian [this] is not good; she said, “Give the fruit at 10 o’ clock.” There used to be coffee, tea, and biscuits, and instead, they now give us fruit: banana, apple, or a pear.

(N6)

In addition to supporting their health, providing nutritious food often increases attendees’ satisfaction with the meals and their interest in learning about eating habits that are relevant to their medical conditions, as the following participants mentioned:

We would like to have more lectures on healthy nutrition and physical activity here… and for fruits to be served instead of pretzels.

(M2)

The second category in this theme refers to ecological issues affected by food and mealtimes in the ADCs. Several such concerns were raised, particularly regarding the use of disposable products at most ADCs, which increased in some centers following the onset of COVID-19. While some centers use disposable items for all meals, others limit their use to breakfast and coffee breaks. Despite the environmental implications, however, most attendees seem to accept the use of disposable cutlery and dishes, not viewing it as a critical issue:

We eat from disposable dishes, everything is disposable… It’s not so healthy, but what can you do? Even before COVID-19, we used to eat with disposable utensils.

(S7)

In such centers, the environmental influences seem to be less acknowledged and less significant for attendees and instructors. Many did not even refer to the ecological aspects, highlighting instead the potential adverse health impacts of using disposable products.

In centers that use reusable cutlery and dishes, participants described mealtimes as having a livelier atmosphere. They suggested that this promotes greater engagement, as diners assist in returning plates after the meal, and those who are more independent can help their peers with serving and cleaning up. The use of reusable dishes creates a sense of permanence and stability, unlike the feeling that often comes with a one-time meal using disposable products. It also fosters a sense of contribution, as handling reusable cutlery and dishes requires more effort, prompting attendees to feel like they should help out in the process.

There are volunteers from the club who serve everyone their dish on a plate. There are always volunteers. There are younger [attendees] and [fewer] young people, so the younger volunteer to serve. There is a spoon, fork, and knife… everything! [At the end of the meal] we put the dishes in the cart, and the employee puts them in the dishwasher.

(E5)

A significant amount of food is discarded after meals, both because of a reluctance among some attendees to eat it and because there is no strategy in place for conserving or redistributing the leftover food (such as letting people take leftovers home or passing them along to another institution that will use them).

There is no variety in the food; it’s the same dishes every week. The food isn’t good, it’s dry, the chicken is unclean, and a lot of food is thrown away uneaten.

(M1)

And what happens if someone doesn’t come? So, they ask about him, but he doesn’t get his food. Even if someone goes out in the middle of the day, they never give him food. Only during COVID-19 did the manager distribute food to someone who was in isolation. If I don’t come, then they don’t bring me food.

(M7)

Discarded food seems to be a source of frustration for attendees, many of whom stated that both the quality of the food and the management of waste need improvement. However, food waste was not related to environmental concerns at any point during the focus groups or interviews—neither attendees nor staff acknowledged these impacts.

The third theme also highlights various aspects of food security affected by eating at the ADC. A primary objective of ADCs is to ensure food security for older adults in the community. Some attendees mentioned that the meals provided at the ADC represent their only opportunity to enjoy a warm meal throughout the day:

I eat [in the ADC] better than I eat at home.

(G2)

Some people come here because of a lack of food at home, some because they are old and cannot cook. Good and healthy food should be given.

(S5)

Concerns about sufficient food at the ADCs were raised as an issue related to food security. Attendees expect meals that not only meet their basic needs but also allow them to eat well and feel satisfied. However, in some centers, the food quantities are so exact that if someone is still hungry after they receive their portion, an additional portion is not always available.

On Sunday, we get two meatballs […] each; if there are any leftovers, then we get extra. Those who want a side dish usually get *Burghul* [shredded wheat grains] or [pasta] flakes. We also get a fresh salad in a real tiny bowl. There is no dessert usually, and dessert can also be a fruit.

(G5)

The chicken is very small. The lunch is too small, not enough. It’s just so that we won’t be hungry until we get home.

(M5)

Attendees expressed feelings of vulnerability related to the food served. These feelings are intensified by the lack of choice in having to eat at the center and can negatively impact quality of life and sense of well-being. Some attendees pack up the hot meal from the center (which is allowed) to eat later at home or even to feed others at home:

Lunch is taken home, but I don’t eat it. I give it to my children.

(R3)

At lunch, everyone gets a portion, but I don’t eat here, so I take [the food] in boxes that I bring from home. I don’t eat here because I eat in the evening. I don’t eat lunch.

(M6)

It appears that in some instances, the food served at the center is used for purposes beyond simply providing nourishment at the center itself. This may indicate additional food insecurity issues that need to be further explored.

## 4. Discussion

The primary objectives of this investigation were to examine the eating and dining experiences of ADC attendees; assess their health and well-being needs; and identify specific strategies to enhance the institutional dining experience, promote health and well-being, and encourage sustainability. Our findings indicate that the effects of eating and dining in ADCs go beyond purely satisfying the nutritional needs of attendees. Our comprehensive analysis reveals three principal themes encompassing individual, social, and environmental dimensions of eating and dining experiences. Based on these dimensions, we propose a series of strategies that ADCs’ management could implement to enrich both the individual and collective dining experiences of their clients while also advancing their health and well-being, as well as the centers’ sustainability. The dining experiences at ADCs can serve as a pivotal element in meeting the individual and social needs of attendees, which in turn can play a crucial role in preventing the decline of their health and fostering their well-being and resilience. The following sections elaborate on the study’s findings in accordance with the objectives and discuss the potential interventions derived from the investigation.

### 4.1. Individual Needs of ADC Attendees Regarding the Mealtime Experience

Our findings highlight the importance of prioritizing attendees’ concerns and needs during dining experiences. As found in previous studies [11], food choice was a theme of major concern among attendees in our research, with some expressing dissatisfaction with the repetitive nature of the menu and many expressing a strong desire for greater influence over menu decisions and to be able to actualize their personal identity through their food choices. This entails tailoring meals to fit attendees’ personal tastes and health conditions and involving them in various aspects of the dining protocol and menu development. Attendees revealed a pronounced desire for recognition and active participation in the decision-making processes in shaping meals at the center. This fits with the healthy placemaking approach, which calls for the active contribution of participants in shaping the environment so as to provide for their unique preferences and needs [20,24]. Given the myriad physical and mental changes associated with aging, including social exclusion [32], diminished self-esteem [33], and a decline in perceived quality of life [34], it is clear that maintaining autonomy, enhancing self-efficacy, and strengthening social connections are essential elements of promoting the mental health of older adults [35,36]. These objectives can be effectively addressed within the context of ADC dining experiences. Adopting strategies such as on-site cooking, as opposed to relying on catered and prepackaged meals, can significantly enrich attendees’ dining experiences. By being engaged in all stages of meal preparation and service—from designing adaptable menus that cater to their preferences (e.g., fresh and varied foods) and dietary requirements, to participating in cooking, serving, and cleaning up—attendees can have more meaningful and positive dining experiences. This not only enhances their enjoyment of the meals themselves but also supports positive aging through mealtime interactions at the ADCs [37]. Likewise, the healthy placemaking paradigm advocates for the active engagement of participants in the creation of their own meaningful environments, which can augment both physical and mental health [21].

### 4.2. Social Needs of ADC Attendees Regarding the Mealtime Experience

From a social standpoint, our findings show that mealtimes at ADCs are vital social junctures, as they provide opportunities for attendees to mingle, forge new bonds, and mitigate feelings of loneliness and boredom. Moreover, attendees often view their eating and dining experiences at ADCs as a gateway to increased social interaction with the wider community, as exemplified by activities including preparing special dishes for groups such as schoolchildren. These observations align with existing literature that has underscored the social dimensions of mealtimes and their potential role in nourishing both the body and the mind [38]. As described in previous studies [14,15], attendees in our research emphasized the positive effects that social interactions with each other and with other community members can have on the mealtime experience and how these interactions can increase their enjoyment of the meal. Additionally, our findings provide further evidence supporting the “aging in place” paradigm, which emphasizes the importance of public places and the interactions that take place in them in promoting social interactions and health among older adults [22,23]. Consequently, we recommend that the ADC coordinators design dining experiences that strengthen the social aspect. This will help meet the need to encourage social connections between older adults and the wider community so as to bolster both personal and community resilience, which is important during both routine times and in emergencies [39,40].

### 4.3. Sustainability, Health, and Environmental Aspects of ADCs’ Mealtimes

Despite attendees’ awareness of aspects such as the use of disposable cutlery and dishes and the discarding of leftover food, they are not concerned about or interested in these aspects from an environmental or sustainability perspective. This corresponds with previous findings that environmental and sustainability issues are primarily a core interest for younger individuals [41]. Viewed through the lens of the healthy placemaking approach, this lack of awareness is unfortunate since, in the long-term, it can negatively affect the sustainability of the attendees’ environment and, in turn, their health [42]. This highlights the need for a broader educational and engagement strategy within ADCs to elevate the knowledge of the importance of environmental sustainability among older adults. Such initiatives could integrate informative sessions and practical activities that demonstrate the impact of sustainable practices on personal and community health, thereby aligning environmental concerns with attendees’ health-oriented values. Such activities could include setting up recycling projects, sustainable gardening workshops for growing vegetables and herbs for the attendees’ use (thereby increasing their consumption of these healthy foods), or compost bins where attendees can deposit food scraps from meals.

The preference for reusable dishes expressed by some attendees indicates a desire to implement practices that are more environmentally friendly, and that also enhance the dining experience by making it feel more authentic and engaging. This issue is intertwined with the broader problem of food waste; as highlighted by attendees’ reports of leftovers being discarded, there is a significant opportunity for ADCs to implement more sustainable food management systems. Furthermore, the discussions around food security underscore the central role that ADC meals play in the daily lives of many attendees, for whom these meals may be their primary source of nutrition. The reliance on these meals, and the dissatisfaction expressed by some attendees regarding insufficient food quantity, highlights the need to ensure that they are not only sufficient in quantity but also balanced and nutritious [43].

### 4.4. Implications for Designing Potential Interventions

Following this study, preliminary results are being used to design follow-up initiatives aiming to affect participants’ experiences and improve the conditions for attendees and ADC staff. This is being conducted in a participatory manner using the “Healthy Placemaking” approach. Initiatives related to improving dining at ADCs should focus on enhancing the health and well-being of attendees and addressing their diverse range of needs [44]. At the individual level, meals need to cater to attendees’ medical needs and dietary restrictions. One strategy could be to periodically assess the health conditions and food preferences of attendees while providing a diverse array of nutritious options that are tailored to various dietary needs (e.g., vegetarian, gluten-free, or low-sodium meals). Additionally, offering educational sessions or resources on healthy eating habits and nutrition, specifically those tailored to conditions such as diabetes or heart disease, could significantly enhance attendees’ knowledge and their ability to make informed food choices. This approach would not only address individual dietary needs but also promote overall health literacy among attendees.

Furthermore, strategies to enhance attendees’ dining experiences can include implementing an on-site cooking concept; allowing for personalized, adaptable menus; and encouraging active participation in meal preparation. Increasing attendees’ ability to personalize their meals could also be achieved through increased interaction with the nutritionists and caregivers who are responsible for menu selection and meal preparation. These actions would greatly enhance attendees’ personal autonomy during meals, positively impacting their health and well-being, as found by previous studies [11]. Another approach could be to redesign the dining area to create a cozy, home-like atmosphere and to cultivate social engagement and familiar comforts through thoughtful décor, mealtime protocols, and shared tasks [45].

ADCs can also address the need for socialization by utilizing round tables to encourage interaction, removing distractions such as mobile phones or televisions, and organizing communal meals and joint culinary activities within the center. Cultural-themed dinners, where attendees take turns preparing dishes from their own cultures and creating networks for attendees to connect outside of ADC hours for shared meals, could help alleviate feelings of boredom and loneliness while strengthening a sense of belonging. Additionally, centers could nurture communal bonds through cooking events in collaboration with local institutions (e.g., schools and workplaces) or by preparing meals for those in need. Such activities would foster a sense of significance and empowerment among participants [46,47]. These approaches correspond to the views expressed by attendees concerning the need for positive social interactions during meals and with existing literature that has stressed the importance of social interactions for positive mealtime experiences and the importance of communal places in promoting health and well-being through positive social interactions [11,14,15,22,23].

While food management systems in ADCs must ensure basic food security for attendees, both the nutritional content and portion sizes should be considered [48]. For individuals who rely on the center’s meals as a key component of their weekly nutrition, it is crucial to offer full and balanced meals. Furthermore, options must be provided for attendees who miss a meal at the center or have to leave early, to deliver food to attendees, or allow them to take it home if they cannot eat on-site. This would ensure that attendees do not go hungry and would also reduce food waste, suggesting a practical approach that aligns with both health promotion and environmental sustainability goals. Another possible strategy to increase food security and reduce food waste is to enable attendees to deliver leftovers to other vulnerable populations in the community. This approach corresponds with ecological issues raised by attendees and with the sustainability outlook of the healthy placemaking approach [42] and can lead to the adoption of sustainable food management practices such as using reusable dishes and minimizing food waste [49].

### 4.5. Study Strengths and Limitations

The findings of this study contribute to existing literature concerning the role of mealtimes and dining experiences in supporting the health and well-being of older adults. Among the study’s strengths is its qualitative design, which utilizes the thematic analysis method. These methods provide deep insights into the lived experiences of ADC attendees. Thus, we were able to capture the complex interplay between individual, social, and environmental factors influencing attendees’ mealtime experiences, obtaining a nuanced understanding of how ADC meals cater to nutritional needs and broader social and psychological functions. Moreover, the study’s utilization of the healthy placemaking approach, which promotes elements such as participant engagement in meal planning and preparation, as well as the cultivation of a sustainable environment, underscores innovative ways to enhance autonomy and satisfaction among this population.

The study is also subject to some limitations. The findings are based on a small sample size and are not generalizable to all ADC settings or broader older adult populations who can choose the time and location of their meals or receive care in models different from the ADCs. While a small sample is consistent with qualitative inquiry, it may limit the diversity of viewpoints and the ability to generate broader social discourse, and so can the small size of some of the focus groups. To mitigate this effect, we used a purposive sampling method to recruit as diverse a sample as possible, but even so, all our participants were from ADCs located in a single region of Israel and our sample consisted of a high prevalence of individuals from North Africa. Additionally, our sample consisted only of active older individuals without locomotor restriction and therefore our findings are not generalizable to less active older individuals. Furthermore, qualitative data, while rich in detail, do not allow for quantifying the impact of the identified themes on health outcomes—a subject that could be addressed in future research. There is also potential for bias in the researchers’ interpretation of participants’ experiences and in participants’ responses, as those who chose to participate may have different views or experiences compared to those who declined or were unable to participate.

## 5. Conclusions

This study provides valuable insights into the eating and dining experiences of attendees at ADCs, revealing a complex matrix of individual, social, and environmental influences shaping these experiences. The findings underscore the importance of ADC meals not only in meeting nutritional needs but also in fostering social interaction, enhancing quality of life, and promoting autonomy and independence among older adults. By implementing a healthy placemaking approach, the study highlights how ADCs can transform dining experiences into opportunities for active participation, community engagement, access to nutritious food, cultural and social connections, and environmental sustainability—all of which ultimately contribute to improved health and well-being [18].

Our research reveals that, while attendees value meals that cater to their specific dietary needs and contribute to their social and emotional well-being, they place less emphasis on the ecological impacts of the dining services. This suggests a potential area for growth in terms of integrating environmental sustainability into the mealtime culture of ADCs, which could enhance the overall sustainability of these vital community institutions.

Future research should aim to broaden our findings through larger-scale studies and the use of quantitative methods to better quantify the impacts and to generalize the results. Moreover, interventions designed to increase awareness, education, and practices related to environmental sustainability in ADC settings should be explored. Such efforts could not only improve the ecological footprint of ADC meal services but also enrich the educational and environmental aspects of these centers, aligning them with broader public health goals. By addressing these comprehensive nutritional, social, psychological, and environmental needs, ADCs can play a pivotal role in supporting the successful aging of attendees, making these centers not only care-oriented facilities but also vibrant, proactive communities that significantly contribute to the health and well-being of older adults.

## Figures and Tables

**Table 1:** Participants and setting characteristics (*n* = 28).

	No. of ADC Attendees	Gender of FG Participants	Sex of Leadership	Heritage/Cultural Affiliation	ADC’s Settlement Area	No. of FG Participants
ADC 1	40	Mixed	Female	Various	Urban	4
ADC 2	40	Female	Female	North Africa	Urban	5
ADC 3	30	Male	Male	Bedouin	Rural	2
ADC 4	14	Male	Male	North Africa	Rural	3
ADC 5	40	Mixed	Female	Various	Urban	5
ADC 6	25	Mixed	Female	India	Rural	4
ADC 7	30	Mixed	Female	North Africa	Urban	5

Notes. FG = Focus group.

**Table 2:** Emerging themes, categories, and codes.

Theme	Categories	Codes
Theme 1: Needs and objectives at the individual level	Positive experiences relating to food at the ADC	Enjoyment derived from the food served and from mealtimes themselves; use of spices.
		Occasional menu variety; exposure to new dishes.
		Freshness of cooked food; fresh fruits and vegetables; preference for food cooked on-site.
		Feeling of being cared for; motivation and ability to consume nutritious food.
	Empowering experiences from meals at the ADC	Having one’s own place.
		Efficacy and independence.
		Autonomy; ability to actively participate in decisions regarding food choices and dining protocols.
	Eating environments that promote warmth and domesticity	Enjoyment of food that appears homemade and from experiencing human contact.
		Seating arrangements; mealtime atmosphere.
Theme 2: Needs and objectives at the social level	Food as a bonding factor that promotes relationships with others	Time to meet and talk; opening of new social circles.
		Sense of belonging; feeling part of a group with joint experiences.
		Reduced loneliness and boredom.
	Community and social engagement	Contributing to others in the community (active residence).
		Being part of the community.
Theme 3: Sustainability, health, and environmental aspects	Expectations concerning a diet that caters to medical and dietary restrictions	Specific dietary adjustments.
		General dietary recommendations.
	Ecological issues	Use of disposable products.
		Use of leftover food.
	Various food security aspects	Having a warm meal during days the ADC is open.
		Having enough food at the ADC.
		Having good-quality food that is nutritionally balanced.

## Data Availability

The data presented in this study are available upon request from the corresponding author due to privacy and confidentiality restrictions.
